# Evaluating the effectiveness of the EU’s approach to the sustainable use of pesticides

**DOI:** 10.1371/journal.pone.0256719

**Published:** 2021-09-16

**Authors:** Florența-Elena Helepciuc, Arpad Todor

**Affiliations:** 1 Institute of Biology Bucharest, Romanian Academy, Bucharest, Romania; 2 National University for Political Studies and Public Administration, Bucharest, Romania; Szechenyi Istvan University: Szechenyi Istvan Egyetem, HUNGARY

## Abstract

By adopting the 2009 "pesticide package," the EU proposed a common approach to limiting the harmful effects of pesticides, promoting Integrated Pest Management, and the progressive replacement of the most dangerous pesticides with low-risk alternatives through a comprehensive but flexible framework for all EU Member States. Each EU Member State had to develop a National Action Plan that would propose measures to achieve the package’s goals. Nevertheless, the choice of actions and indicators remained to be established at the national level. A series of recent evaluations of how Directive 2009/128/EC of the European Parliament and the Council on the Sustainable Use of Pesticides Directive (SUD), a central piece of the "pesticide package," was implemented showed limited success in achieving its goals. Aiming to explain these failures, we compare the National Action Plans eight EU Member States adopted after the SUD. We assess the degree to which the countries’ proposed measures and indicators would achieve the Directive’s three overarching objectives (reduce risks and impact; promote Integrated Pest Management; promote approaches and techniques to reduce reliance on pesticides). We develop the comparative analysis along three dimensions: the promotion of measures to achieve SUD’s three goals; the evolution of the pre-and post-Directive action plans of some of the old EU Member States; and the differences between old and the new EU Member States. The comparison along ten variables shows that the SUD had a minimal effect in homogenizing different states’ approaches to develop their National Action Plans to systematically treat problems, propose measures, and timetables for implementation and indicators. Given that the overall effect in generating a common EU approach to raise the sustainability of pesticide use and agriculture, in general, was still limited, as no common measures, indicators, or process to planning were identified, we discuss some suggestions to improve the situation.

## Introduction

Despite the ever-increasing awareness of the harmful effects of pesticides and continuous efforts to eliminate the most damaging substances and to promote low-risk alternatives [1], the fact is that modern agriculture is, for the time being, reliant on synthetic pesticides [2]. The progress in the European Union’s (EU) regulatory capacity in the last two decades had allowed it to develop a comprehensive system for pesticide authorization, especially plant protection products (PPP). The EU defines pesticides as "something that prevents, destroys, or controls a harmful organism (’pest’) or disease, or protects plants or plant products during production, storage, and transport (herbicides, fungicides, insecticides, acaricides, nematicides, molluscicides, rodenticides, growth regulators, repellents and biocides)." PPPs are defined as ‴pesticides’ that protect crops or desirable or useful plants" [3]. With the 2006 Thematic Strategy on the Sustainable Use of Pesticides and the 2009 adoption of the "pesticide package," the EU took some bold steps toward attaining the sustainable use of pesticides [4]. The other two goals of *Directive 2009/128/EC of the European Parliament and of the Council of Oct 21 2009 establishing a framework for Community action to achieve the sustainable use of pesticides* (Sustainable Use Directive or SUD), focused on promoting alternative approaches or techniques to reduce dependency on the use of pesticides, mainly by increasing the availability and usage of low-risk pesticides and the development of organic agriculture [5] and Integrated Pest Management (IPM) [6]. The European Commission (EC) defines IPM as the "careful consideration of all available plant protection methods and subsequent integration of appropriate measures that discourage the development of populations of harmful organisms and keep the use of plant protection products and other forms of intervention to levels that are economically and ecologically justified and reduce or minimize risks to human health and the environment." IPM implementation is essential as "integrated strategies showed a decrease in the use of both pesticides and nitrogen fertilizers; they consumed less energy and were frequently more energy efficient. Integrated systems, therefore, appeared as the best compromise in sustainability trade-offs" [7].

Part of the "pesticide package," *Regulation (EC) No 1107/2009 of the European Parliament and of the Council of Oct 21*, *2009*, *concerning the placing of plant protection products on the market* [8] introduced a series of derogations for low-risk active substances to create a more relaxed regulatory regime in comparison with that applicable for synthetic-chemical pesticides. The efforts to promote the availability and use of low-risk pesticides was likewise supported by the European Parliament’s Declaration on low-risk pesticides of biological origin (2016/2903(RSP)) [9], which stressed the need to accelerate the availability and use of low-risk pesticides to:

"Accelerate the evaluation, authorization, registration, and monitoring of the use of low-risk plant protection products of biological origin while maintaining risk assessment at a high level;

Include the use of low-risk pesticides of biological origin in their national action plans on the protection of the environment and of human health;

Encourages the Member States to exchange information and good practices deriving from research results into pest control, enabling the provision of alternative solutions viable in environmental, health, and economic terms."

Furthermore, the SUD’s efficient application is vital as Eurostat data shows that pesticides sales across the EU have not decreased after 2011 when the SUD should have been fully transposed [10]. The pesticide sales per agricultural land are slightly increasing in countries like France, Hungary, and Poland and remain stable in the rest of our analysis’ countries.

Unlike EU treaties, regulations, or decisions, EU Directives propose a series of results but leaves the means to achieve those results to the Member States. The Sustainable Use Directive, adopted in October 2009, required the Member States to draw up National Action Plans (NAPs) to implement the Directive by November 2012. To achieve the objective of the SUD, each NAP (under Article 4) needed to: (a) propose measurable goals, targets, and indicators to decrease the potential and proved effects of pesticide use on humans and the environment; (b) stimulate the expansion of IPM and alternative approaches or methods to reduce reliance on pesticides [4]. The common elements required by the SUD are: timetables and targets; provisions regarding the information on the risks of pesticides to people involved in their application; provisions regarding the application of IPM by all professional users by 2014; and crop and sector-specific guidelines [11]. The action plans’ detailed structure and the institutional measures to put them into practice were left to the individual Member States.

Furthermore, while the SUD Directive called at Article 15 for the establishment of EU-level harmonized risk indicators that would allow a comparison of progress among the EU Member States, this process stalled and only restarted in 2019 through the adoption of the 2019/782/EC Directive, which amended the SUD Directive [12]. A 2014 evaluation presented some of the most influential Pesticides Risk Indicators to be CEPI (Belgium), Field margins (Germany), Hair (EU) Pesticides UK (UK), PRIME (Canada), SYNOPS GIS, SYNOPS TREND (Germany) [13]. Thus, there has been no harmonized indicator to objectively compare different EU Member States’ progress during the first NAPs were designed and implemented. Only in 2019, the European Commission calculated the evolution of two Harmonised Risk Indicators (HRI) retrospectively. HRI 1 measures the use and risk of pesticides and decreased by 17% from 2011 to 2017 and stagnated afterward. HRI 2 measures the evolution of emergency authorizations and shows an overall increase of 56% from 2011.

A first evaluation of the SUD Directive implementation was based on a questionnaire completed by the Member States in 2016. The report [11] was also sent to the European Parliament by the European Commission in a different format [5]. The EC’s evaluation’s main focus was on whether the NAPs proposed improvements in areas such as the inspection of spraying equipment, training and certification procedures, the prohibition of aerial spraying; decreasing poisoning from PPP; and the protection of the aquatic environment and drinking water. Instead, little attention was paid to the efforts to achieve the SUD’s second and third goals. Overall, while this evaluation was generally positive, it mentioned the limited progress achieved in some areas.

Four years later, the 2020 European Court of Auditors’ report underlined the limited progress in measuring and reducing pesticide risks in the EU. EC’s limited monitoring capacities caused this, along with the limited incentives for farmers to implement IPM [14]. The 2020 European Court of Auditors’ report also underlined the few available low-risk plant protection products (16 out of 487 substances, only 3%) [15]. Similarly, a recent evaluation by the RISE Foundation showed that the EU’s strategy to mitigate risks related to PPP and the promotion of IPM had not progressed sufficiently. IPM is not implemented comprehensively, and "risk assessments for PPPs are not fulfilling expectations, timetables for product approvals are not respected due to under-resourced regulations and lack of clear targets, data on current PPP use is inadequate" [15]. In a recent chapter focusing on Romania’s case, we show the limited progress achieved in agriculture’s sustainability due to limited implementation of IPM, low-risk alternatives to synthetic pesticides, and organic agriculture [16].

Given the limited positive evolutions shown by the two HRI, the Commission pledged to increase its efforts to press EU MS to implement "rules on pesticides by Member States (including launching infringement actions if necessary); ensure the full implementation of Integrated Pest Management (IPM)." Another pledge aimed to link the Common Agricultural Policy (CAP) with the progress of pesticides and to evaluate and revise the SUD (possibly by including mandatory targets [17]. Overall, the European Commission had "set the ambitious targets to reduce the use and risk of chemical pesticides (based on HRI 1), and the use of more hazardous pesticides, the so-called candidates for substitutions by 50% by 2030, under the Farm to Fork Strategy" [17]. The Fram to Fork and the Biodiversity strategies are part of the European Green Deal. The European Commission also aims to increase the environmentally-friendly approach to agriculture with "25% of total farmland being used for organic farming by 2030" and a decrease in the use of fertilizers [18].

Other authors also show that while the adoption of IPM principles is compulsory in the EU, given the considerable variation among EU Member State’s commitment and the limited development of crop-specific guidelines, their implementation has limited effectiveness. The widespread adoption of IPM in the EU is also hindered by internal challenges (finding research funds and limited expertise, knowledge transfer at all levels, and networking) and external issues (climate change, development of pesticide resistance) [19]. A significant limitation to a more rapid introduction of IPM rests in the uncoordinated efforts in biocontrol research and innovation in tackling the most critical pests [1]. While IPM does not necessarily entail supplementary costs, individual farmers may be reluctant to adopt IPM due to lack of interest, technical problems or difficulties in changing habits [6], or lack of support from their value chain partners [20]. Thus, national-level efforts to promote the introduction of IPM are crucial. Also, the strategy of pesticide withdrawal needs to be correlated with the implementation of IPM. A study focusing on the challenges in decreasing pesticide use in maize production while maintaining competitiveness revealed the difficulties in reducing pesticide use. Most barriers come from the need to use hybrids, crop rotation, biocontrol solutions, IPM, optimized pesticide application, the limits posed by farm organization, and the limited knowledge among farmers [21].

In this article, we aim to contribute to the explanation of the observed limiting success in achieving the aims of the SUD by performing the first comprehensive analysis of the content of the NAPs of five old Member States (Denmark, Germany, the Netherlands, France, and the UK) and three new Member States (Romania, Poland, and Hungary). While they belong to different authorization zones for PPPs according to Regulation EC 1107/2009, the SUD 2009/128/EC does not include specific requirements for states, depending on the zone to which they belong. While the NAPs do not necessarily present an accurate picture of the measures taken after the plan was adopted, they represent the best-case scenario proposed by each MS. Their comparative analysis offers thus an in-depth view of the EU Member States’ differences in the level of ambition and planning their actions. Unlike the Commission’s evaluation, based on questionnaires completed by the EU MS, in this article, we are directly analyzing the NAPs and thus avoid the biases caused by the tendency of each MS to present measures in the best light. The 2017 evaluation focuses on limiting the negative effects of pesticides but offers no comparative assessment and no cross-temporal comparison. These reports, however, focused only on whether the Member States’ NAPs covered specific technical requirements. Our study goes to more advanced points than the 2017 reports in that we analyze the comprehensiveness of different sections or whether the objectives of the NAPs had indicators and timetables. Our study fits within the Europeanisation of public policies and compliance with EU regulations literature. It is also part of the wider research area of compliance with international institutions [22]. Thus our findings on the EU’s capacity to generate policy change in the EU MS could be meaningful to a broader audience interested in the mechanism through which national states adapt their policies during their integration in the international regulatory regime.

To allow us a cross-temporal analysis, we build on the analytic structure proposed for the first comparison of NAPs designed for five Member States by Barzman and Dachbrodt-Saaydeh [23] before Directive 2009/128/EC and focus our analysis on three research questions:

RQ1: To what extent did the EU Member States NAP’s respect the SUD obligations to propose quantifiable objectives, targets, measures, and indicators to achieve the three goals of the SUD: (1) reduce risks and impacts of pesticide use on human health and the environment; (2) encourage the development and introduction of Integrated Pest Management, and (3) propose alternative approaches or techniques to reduce dependency on pesticides?
RQ2: What are the effects of SUD 2009/128/EC on the NAPs of the countries that voluntarily developed them before adopting the Directive?
RQ3: To what extent did SUD 2009/128/EC drive homogenization in the approach to sustainable use of pesticides among the old and new Member States?

## Materials and methods

For this analysis, we have studied the NAPs [24] of the eight EU Member States listed above and two evaluations of the European Commission [5, 10]. To analyze both the cross-sectional (RQ1) and the cross-temporal (RQ2) differences, we had started by using the analytic scheme and data by Barzman and Dachbrodt-Saaydeh [23] in their 2011 analysis (before Directive 2009/128/EC made NAPs compulsory). They analyzed the voluntary NAPs by Denmark, Germany, the Netherlands, France, and the United Kingdom. Afterward, we have analyzed the eight NAPs developed after the SUD adoption and the European Commission’s 2017 evaluation. Using the approach proposed by Barzman and Dachbrodt-Saaydeh, we have examined the NAPs on the following dimensions: the volume-reducing approach (only an implicit goal of the SUD Directive), treatment frequency, risks and impact, stakeholder involvement and research and extension, all part of the first goal of the SUD (see the synthetic results in Table 3 and the extended one in Table 1). On the SUD’s first goal, we have also analyzed the NAPs in search of measures aiming to reduce pesticide use in specific areas and information and awareness-raising towards the public on PPP usage and effects (see results in the first two columns in Table 1). We have also analyzed the measures to promote the adoption of IPM (SUD second goal) and the measures to promote the usage of low-risk products and organic farming (SUD third goal) (results in Table 2). The European Commission defines organic farming as "an agricultural method that aims to produce food using natural substances and processes" [25]. For each of these measures, we have looked at the following elements: whether the dimension is treated systematically and how many times it is mentioned in the text; whether the dimension is discussed in a dedicated section of the NAP; whether there are measures that can be operationalized; whether there is a clear timeline for the measures; and whether there are quantifiable methods or indicators proposed to assess progress. We have comprehensively analyzed all the documents and also performed a different search using keywords for each dimension. The third research question (RQ3) is approached by comparing the NAPs of the five old EU Member States with the NAPs of the three new Member States across all dimensions.

**Table 1:** A comparative survey of EU comprehensive analysis, and the National Action Plans of Denmark, Germany, the Netherlands, France, the UK, Romania, Hungary, and Poland along the following dimensions: Volume reduction targets, treatment frequency and efficacy, risk reduction, impact reduction, stakeholder involvement, and research and extension.

NAPs	Volume reduction targets (implicit SUD first goal)	SUD first goal: Treatment frequency and efficacy	SUD first goal: Risk reduction and risk indicators	SUD first goal: Impact reduction	SUD first goal: Stakeholder involvement and research and extension
**EC 2017 evaluation—All EU MS** [11]	9 MS use reduction objectives	No systematic discussion. Reference to the Danish Treatment Frequency Index (TFI). Reference to Germany, as the only MS to use a yield increase indicator that would measure the yield increase following PPP application	21 MS established risk reduction objectives, but "only five Member State NAPs set high-level measurable targets, of which four relate to risk reduction and one to use reduction. The Netherlands also has measurable targets for risk reduction, although these are established outside their NAP" [11]	No systematic evaluation of NAPs application achievements of the Directive 2009/128/EC of the European Parliament and of the Council on the sustainable use of pesticides’ achievements. Reference to the Danish Pesticide Load Indicator (PLI) as a best practice	One mention of Netherlands as a best practice example
**Before 2009** **Denmark** **2017 NAP (2017–2021) Pesticide Strategy 2013–2015**	Focus on use reduction	Systematic treatment and a quantitatively measurable target through the Treatment Frequency Index (TFI) ("how many times on average a conventionally utilized agricultural area can be sprayed with the amount of pesticides sold, and applied in standard dosages")	IPM and the "green growth" economic policy were translated into crop protection sub-policies via a closed process among five ministries. To avoid farmer rejection of compulsory measures, the government covers up to 80% of costs incurred by farmers for advisory support on Integrated Pest Management.	Treatment Frequency Index was replaced with "pesticide impact index" (Pesticide Load (PL) to be reached by 2013 [23].	Initially approaching NAP through binding legislation but later implemented a multi-stakeholder consultation approach for developing the NAP
	Change of focus on risk reduction, given that use reduction, proved not to be effective in risk reduction given the vast differences among synthetic PPP	Systematic treatment and a quantitatively measurable target through the Treatment Frequency Index (TFI)– 50% reduction over ten years in combination with the new "pesticide impact index" (factors in "pesticide use, the extent of non-sprayed areas and the pesticide burden on health and the environment.") ("green growth" policy)	According to COM’s evaluation, one of MS in which "substantial progress towards the achievement of their risk reduction targets could be demonstrated." [11] Clearly defined target for risk reduction of 40% (Pesticide Load Indicator) combined with the employment of pesticides taxes. COM’s evaluation shows that the targets have been achieved by 2015.	"Overall objective to achieve a 40% reduction in pesticide loads on human health, nature, and groundwater by the end of 2015" [26].	Research and extension efforts focused on the optimization of existing systems and optimizing the use of pesticides.
**Before 2009** **Germany** **2012 NAP**	No mention		Environmental risks associated with synthetic PPP reduced by over 50% during the 1987–2007 period, while volumes have increased [11]. 25% risk reduction by 2020 aimed in the 2009 NAP.	No specific mention	Starting in 2002, proposed a new approach based on multi-stakeholder consultations in drafting the reduction program. NGO PAN Germany’s pressure was essential in imposing a quantitative target in the 2009 NAP.
	No mention	General mentions, but no specific measures or targets [32]	One of MS which in "substantial progress towards the achievement of their risk reduction targets could be demonstrated" according to COM’s evaluation aimed a "20% reduction in the environmental risks associated with pesticide use by 2018, and a 30% reduction in risk by 2023, compared to the 1996–2005 period [11]."	Measures to reduce the pollution impact of plant protection products on pollinating insects	Nine mentions, but no systematic approach Most responsibilities are places at the Länder level that produce an index for advisory capacity necessary for NAP implementation.
**Before 2009** **the Netherlands** **2012 NAP**	No mention	No mention	The 2003 National Agreement on Crop Protection aimed at "a 95% reduction in the environmental impact by 2010 relative to 1998, as measured by the ratio of predicted exposure concentration on water organisms to the no-effect concentration [23]." Achieved a pesticide **impact reduction** on the surface and drinking water by 85% and 75% by 2010 compared to 1998.		The systematic use of the "polder model" implies the coordination of diverse stakeholders around large initiatives. ("ministries of agriculture and the environment, farmer organizations, the pesticide industry and distributors, water boards and water companies, input suppliers and NGOs.")
	No mention	No mention	One of MS in which "substantial progress towards the achievement of their risk reduction targets could be demonstrated" ((Environmental Indicator for Pesticides) according to COM’s evaluation. The Netherlands also has measurable targets for risk reduction, although these are established outside their NAP [11].	Employment of HAIR2010 as an indicator "to determine trends in the environmental impact of pesticide use on, for instance, surface water," No concrete actions or goals associated to this indicator.	Encouragement of farmer communication with neighbors and residents regarding pesticides usage. No discussion of research
**Before 2009** **France** **2015 NAP**	NAP proposed a 50% volume reduction, but the use increased by 5%. Measures to remedy the situation through the adoption of innovative techniques [23].	50% reduction in treatment frequency aim	2006–2009 required the development of an environmental risk indicator, but its finalization was postponed until 2012 [23].	No specific mention	In 2007, however, the government-launched ’Grenelle de l’Environnement’ by involving stakeholders in consultations and elaboration of the Ecophyto 2018 plan
	One of the core seven principles of the NAP: "a 25% reduction by 2020, based primarily on optimizing production systems through the transfer and dissemination of currently available solutions;—a 50% reduction by 2025 [27]."	"Roll-out of use indicators: Unit dose number (NODU), active substance quantity (QSA), treatment frequency index (IFT). IFT will be retained as a support and measurement tool for a reduction in the use of plant protection products at the farm and territorial level [27]."	Measure to "expand multidisciplinary research on the environmental and health risks and impacts on the air, soil, and water pollution linked to plant protection products." The proposed measure to establish timetables and targets to achieve use reduction aiming also to achieve a risk reduction No risk indicator. Aim to calculate "Calculate impact indicators: product toxicity, health impacts, impact on biodiversity, bio-pest resistance [27]."	Measures to stimulate research on methods to decrease the impact of PPP on the environment and usage of alternatives	Measures proposed through the National Strategy for Research and Innovation to identify the priority actions to achieve the four core objectives: promote IPM, limit dependence on synthetic PPP; reduce the risks and impacts; "identify and address socio-technical and economic barriers to a shift in practices and support changes in practices and sectors [27]."
**Before 2009** **UK** **2013 NAP**	No mention	No mention	No mention	No mention	The Pesticide Forum is a multi-stakeholder group set up in 1996. It brings together representatives of environmental, conservation, and consumer interests, as well as from farming and the pesticide industry, and oversees and monitors the six action plans and advises the government. Other non-governmental stakeholder’s initiatives can obtain significant responsibilities.
	No mention	No mention	Proposed measures, but no quantitative targets. Proposed obligations of pesticide users to reduce risks of using pesticides by "adopting an integrated approach as described in the Directive, drawing on all available techniques to tackle pest; complying with all relevant regulations and record-keeping requirements for pesticides; complying with any Codes of Practice and following guidance–for using pesticides appropriate to the local situation; supporting the measures in this plan relevant to their sector [4]." No risk indicator	NAP’s objective to reduce pesticides’ risks and impact and to encourage IPM and alternative approaches. Mention of the role of users in achieving the goals	The NAP has been developed through stakeholder consultation. Future consultations, review, and annual reporting would be carried through the UK Pesticides Forum Presentation of the list of programs that: "capture any human health effects of pesticides Pesticide Users Health Survey (Health and Safety Laboratory); Human Health Enquiry & Incident Survey (HHEIS) (via pesticide approval holders); The Health and Occupation Reporting network (THOR) at the University of Manchester; UK Hospital Episode Data through the NHS; Pesticide Incidents Appraisal Panel (PIAP); National Poisons Information Service [26]. "
**Romania 2013 NAP**	Mention of the need to decrease reliance on chemical PPP, but no concrete measures	Mention, but no concrete proposals	Measures proposed regarding reducing risks for professional users and distributors of PPPs, residents, and passers-by; protecting biodiversity by limiting PPP risk of pollution; regulating storage, marketing, and usage of PPP [28]; risk related to chemical exposure [4] but without a clear operationalization, timelines or quantitative evaluation. No risk indicators	Reducing the impact of PPP on human health and the environment except lowering the impact of PPP on pollinating insects. The measures are minimal: monitoring intoxication cases, and one awareness-raising session with farmer session/year	General mention—"all stakeholders in the area must have access to training on IPM" [28] Research—No mention
**Hungary 2012 NAP**	No mention, but detailed analysis of pesticides usage	No mention	Only about aerial spraying, but no specific measures [29] No risk indicators	Detailed analysis of measures to reduce the impact of PPP on the environment	Aim to evolve, create a Development of National Plant Protection Programs for Research and Innovation to ensure both classical PPP and "non-chemical treatment-based and alternative pest management to maintain and promote the competitiveness of agricultural holdings," minimizing synthetic PPP use, and allowing for adaptation of pests patterns [29].
**Poland 2013 NAP**	No mention	Treatment record-holding obligations for users and determining minimal safety distances from various areas "(roads, water intake protection zones, surface waters, apiaries, nature reserves);" application of PPP under optimal weather conditions for their efficiency [30]; a systematic approach to testing PPPs’ efficacy; supervising the technical efficiency testing for sprayers by the State Plant Health and Seed Inspection Service; systematic efficacy testing of PPP and the functioning of bodies that oversee testing according to international standards [30].	Systematic treatment of risk measures, but the development of a set of indicators set for the 2013–2015 period [30].	Nine mentions, but no specific indicators	No mention of stakeholders’ consultation Research—"Measure 9. Use of research for Integrated Pest Management and reduction of risk associated with the use of plant protection products."

**Table 2:** A comparative survey of EU comprehensive analysis, and the National Action Plans of Denmark, Germany, the Netherlands, France, the UK, Romania, Hungary, and Poland along the following dimensions: Reduction of pesticide use in specific areas, support for Integrated Pest Management, support for the usage of low-risk products, support for organic farming, information, and awareness-raising towards the public on PPP usage and effects.

NAPs	SUD first goal: Reduction of pesticide use in specific areas	SUD first goal: Information and awareness-raising towards the public on PPP usage and effects	SUD second goal: promoting Integrated Pest Management	SUD third goal: Low-risk alternatives	SUD third goal: Organic farming
**EC 2017 evaluation—All EU MS** [11]	EU-wide measures adopted: measures to minimize PPP in public areas (26 MS); specific use reduction targets for public areas (four MS); measures dealing with recently treated areas in terms of protecting agricultural workers in treated areas (four MS); general prohibition of pesticide use in public areas (four MS). Five MS set high-level measurable targets, with 4 having risk reduction targets (Belgium, Denmark, Greece, and Germany) and one (France) having a use reduction target [11].	EU-wide measures adopted: website (28 MS); farmers’ obligation to inform neighbors and local residents before PPP application (six MS—Spain, Croatia, Sweden, Netherlands, Hungary, Malta); mandatory for the spray operator/landowner to erect signs at the location to be treated (2 MS).	EU-wide measures adopted: a mix of tools to support IPM (28 MS); publicly funded systems for forecasting, warning, and early diagnosis for pest and disease control (four MS); established networks of IPM demonstration farms to develop and disseminate IPM techniques (four MS).	Analysis of the pesticides package (15 basic substances approved), but no concrete data on MS measures.	All MS use EU funds to support organic farming, but no quantitative evaluation is provided.
**Denmark 2017 NAP (2017–2021) Pesticide Strategy 2013–2015**	Starting 1998, steep measures have been implemented in several areas: pesticide use on railways has been reduced by 50% using spot spraying; quota for pesticide on each golf course. A 90% reduction in public areas between 1995 and 2013 [31]. In the Pesticides Strategy 2013–2016, the objective regarding pesticide use was for the Pesticide Load Indicator (PLI), which is based on sales data, to fall to 1.96 in 2015, which corresponds to a 40% reduction relative to the level calculated for 2011 [31]. PLI replaces *treatment frequency index (TFI)* as the core indicator	Awareness campaigns regarding regulations (with a focus on distributors) coupled with increased controls (goal: decrease non-compliance from 30% to 5% by 2019); the "Think before you spray program" is presented a good practice example in the COM evaluation (The campaign gave advice on alternative methods for dealing with weeds and pests and promoted the use of Ready-To-Use pesticides) [11].	Detailed and systematic set of measures. Therefore we will: Set up a one-year IPM task force; establish a Partnership for Spraying and Precision Technology; launch a follow-up survey in 2020 of developments in herbicide resistance since the period; prepare an action plan based on the IPM principles to address resistance; continue to develop, test and provide advice about IPM tools that prevent the development of resistance and contribute to the implementation of the IPM principles in agriculture and horticulture, e.g., in locally-embedded projects; continue efforts to train professionals at farms and horticulture, greenhouses, nurseries, etc., as well as teachers and consultants, in the IPM principles. (p.21-22)	Proposed measures: promote basic substance approval at the EU level, promoted their usage at the national level; adapt data requirements for biopesticides to allow wider usage for organic farming and conventional farming; subsidize access to biopesticides and authorization costs for biopesticides usable in organic farming. [7]	Subsidies to organic pesticides as a means to encourage organic farming
**Germany 2012 NAP**	A country in which "there is a general prohibition of pesticide use in public areas," with possible regulations when biological controls or low-risk PPP are not available.	Mention MS’s obligation under the Directive and how it is fulfilled by using websites of the central at Federal and Länder level [32].	IPM plays a central role in the strategy, with 90 mentions. Drawing 100% crop-specific guidelines by 2018 to be developed by grower organizations "so as to ensure their relevance and implementation." The 2017 COM evaluation revealed that "to date, growers have not developed such guidelines." Subsidies through CAP funds for employing IPM.	NAP’s goal is to move agriculture towards alternative solutions, low-risk products being important. No measures or targets are discussed	Fifty-seven mentions within the NAP. The specific chapter "6.1.1 Promoting the initial and further development of measures for risk reduction in plant protection (integrated plant protection and organic farming)" with a clear target: "increase in the proportion of agricultural area on which work is performed according to the directive on organic farming (National Sustainability Strategy) 20% of the agricultural and horticultural area [32]."
**the Netherlands 2015 NAP**	Prohibition of pesticide usage on hard surfaces.	The obligation of farmers to inform neighbors and residents before the pesticide application [11]. COM’s evaluation presents the "Green Deal" as the best collaboration between stakeholders in addressing specific issues. Five such deals are mentioned. The awareness-raising campaigns focus on minimizing herbicide usage in nonagricultural sites.	Specific chapter in the NAP for IPM, a general discussion about the importance of knowledge and methods dissemination with the aim: "2014, all professional users will be applying the principles of Integrated Pest Management." Mentioned in COM’s evaluation as a good practice case with a mandatory recording of all IPM measures by farmers ("crop rotation, use of resistant varieties, biological, physical and non-chemical methods, selection of pesticides based on risks for environment and humans, monitoring of harmful organisms, use of warning and forecasting systems and resistance management").	One indirect mention that the pesticides sellers should provide information on low-risk alternatives	No mention
**France 2015 NAP**	No direct mention. Indirectly discussed in the section on increasing the monitoring capacity of contamination with PPP and public exposure.	Systematic treatment of one of the seven principles—Create a positive ethos by mobilizing all stakeholders by promoting awareness-raising of the general public on how the measures will "shift within French agriculture towards systems that offer good economic, environmental and social performance." See RISK "Expand multidisciplinary research on the environmental and health risks and impacts of the air, soil, and water pollution linked to plant protection products." And increase awareness about these risks	Systematic treatment (26 mentions), goal to support 30.000 farms in introducing IMP	Promotion of use in the larger context of agroecology: "low-plant protection product practices will depend in large part on providing support to 30 000 holdings as they move over to agroecological systems that make little use of plant protection products [27]."	NAP is a key instrument in a more comprehensive agroecological project for transforming agriculture. Frequent references and detailed goals: "expand to 3000 the DEPHY (Network for demonstration, trial and reference production for low-plant protection product use systems) farms (from 1900). Stimulation of organic farming for field crops and crop diversification
**UK 2013 NAP**	"The UK did not provide information in this area. However, most of the NAPs set no specific use reduction targets for public areas." [11] Mention of the need to "take ’all reasonable precautions to protect or avoid endangering human health when using, storing and handling pesticides" and the need to "confine pesticide applications to the target areas" [33].	Special section–a discussion of website use, the information dissemination by the monitoring system, and regulatory measures regarding the information the PPP package should contain.	Specific chapter, but no specific measures, indicators, or funding in the area	General mention on advantages and disadvantages of biopesticides and measures to stimulate their usage (p. 26) through research and development and a special Biopesticides Scheme. (£2.1 million invested in research; ten biopesticide active substances approved since the scheme started in 2006)	Mention of the Organic Entry Level Scheme as a subsidy program for farmers to convert to organic methods.
**Romania NAP 2013**	Description of legislation without an evaluation of its implementation and concrete measures	One of the two MS with planned measures for public information	A website with general information and aim to increase its usage by farmers	Measures for the reorganization of the approval system for the plant protection products but no concrete measures for low-risk products	No mention
**Hungary NAP 2012**	No distinct mention	General mention encouraging "communication and dissemination of information among the general public [29].” COM’s evaluation—Obligation of farmers to inform neighbors and residents before the pesticide application [11].	Comprehensive analysis but not quantitative targets (mention for the need for future development)	Stressing the importance of promoting the use of low-risk plant protection products, especially for low quantity crops, increasing availability to non-professional users and in protected areas	Highly encouraged, especially in environmentally vulnerable areas, but no concrete measures. Detailed measures to spread knowledge and to offer support about ecological farming
**Poland NAP 2013**	No mention in NAP. Mentioned in the COM’s evaluation as a country where "there is a general prohibition of pesticide use in public areas," without derogation.	Raising public awareness on PPP through information campaigns and change in curricula of agricultural high schools and colleges	Measures to gather information (annual survey with 1500 pesticide users and every five years with 60.000 users) to assess the implementation of the eight IPM principles. And creating a decision support system for farmers	Proposed measures to promote usage and link with IPM to decrease dependency on chemical PPP	Comprehensive approach linked with the development of biocontrol and a warning system

**Table 3:** Comparative synthetic investigation of the National Action Plans of Denmark, Germany, the Netherlands, France, the UK, Romania, Hungary, and Poland (synthetic data from Tables 1 and 2).

	SUD goal 1							SUD goal 2	SUD goal 3		Cumulative +
NAPs	Volume reduction targets	Treatment frequency and efficacy	Risk reduction and risk indicators	Impact reduction	Stakeholder involvement and research and extension	Reduction of pesticide use in specific areas	Information and awareness-raising towards the public on PPP usage and effects	Integrated Pest Management	Low-risk alternatives	Organic farming	
**Denmark 2012 NAP (2017–2021) Pesticide Strategy 2013–2015**	++	++++	++++	++++	+++	++++	++++	++++	++++	++++	37
**Germany 2012 NAP**	-	+	++++	+++	++	++++	++	++++	++	++++	26
**The Netherlands 2012 NAP**	_-	-	++++	++++	++	++++	+++	++++	++	++++	27
**France 2015 NAP**	++++	++++	+++	+++	++++	+	++++	++++	++++	++++	34
**UK 2013 NAP**	-	-	+++	++	++++	+	++	+	+++	++++	21
**Romania 2013 NAP**	+	+	++	+++	+	+	++	+++	++	-	11
**Hungary 2012 NAP**	+	-	+	++	+++	-	++	+++	++	++	16
**Poland 2013 NAP**	-	++	+++	+++	+	++++	+++	+++	++	+++	23

No mention or treatment; + Mention; ++ Systematic treatment and measures; +++ Measures and timetable; ++++ Measures, timetable and indicators (achieving all the requirements of the SUD)

## Results and discussion

Regarding the first research question, the eight countries’ NAPs contain references to encourage IPM (second goal), low-risk products, and organic farming (third goal). Instead, the proposed measures and emphasis vary widely. IPM plays a central role in the drive towards agricultural sustainability in Germany. Also, France has developed the DEPHY network that included experimental farms (DEPHY FERME) and experiments with cropping schemes aiming to reduce reliance on chemical PPPs [34] and an online platform dedicated to IPM users. France also set the quantifiable goal to support 30.000 farms in introducing IPM. Denmark’s proposed measures show that the implementation of IPM is only in the beginning. Instead, the possible steps to promote IPM are only discussed in the Dutch and UK NAPs. While Hungary’s NAP comprehensively discusses the importance of IPM but proposes no measures, the Polish and Romanian NAPs advance measures for information gathering and dissemination regarding IPM. Nevertheless, no concrete steps to implement IPM are present.

Measures to promote SUD’s third goal by promoting low-risk pesticides (first dimension) are discussed to decrease reliance on synthetic pesticides, but without concrete targets (Germany, the Netherlands). In contrast, there are measures to increase their usage (Hungary, Poland), while others treat them as central to a new approach to agriculture (Denmark and France). For example, Denmark focuses on a detailed set of measures to promote low-risk products. All eight NAPs support organic farming (the second dimension of SUD’s third goal). Some propose comprehensive measures to stimulate it (Germany, the Netherlands, France, and Poland). Other NAPs mention steps to encourage it (Denmark, UK, Hungary), while Romania’s NAP does not discuss it.

Regarding our second research question, the effects of Directive 2009/128/EC on those countries that had already voluntarily developed NAPs, the data in Table 1 shows that, in general, all countries maintained their approach in structuring their post-SUD NAP. The UK emphasized stakeholder consultation but avoided proposing quantifiable targets with exact timetables. On the other hand, France has continued to emphasize using indicators and clear targets for volume, treatment frequency, and risk reduction. The Netherlands has continued to employ a more comprehensive measurement to capture the pesticides’ overall impact on the environment. Denmark has changed its focus from quantity reduction to risk and impact reduction and shifted from the quantitative treatment frequency index (TFI) to the use of Pesticide Load based on”three sub-indicators for human health, ecotoxicology, and environmental fate, respectively… PL does not consider the actual exposure, i.e. it reflects the relative risks associated with the use of pesticides. Besides using PL for monitoring the yearly trend in pesticide use and load, the PL was also used for setting up a new pesticide tax scheme and for setting quantitative reduction targets.” [35] Comparing the changes from the pre to post-SUD NAPs reveals a continuation of putting more emphasis on risk reduction and impact reduction. Also, in the post-2009 NAPs of these countries’ stakeholder consultation are treated less exhaustively, as, in most countries, consultation has become a routine.

All states had to reduce pesticide use in specific areas (a compulsory section for all NAPs related to goal 1 of the SUD). Still, their treatment of the actions varies significantly. Germany and Poland have strict prohibitions on PPP use in public areas, while France, the UK, and Hungary do not discuss this issue in their NAP. Romania’s NAP presents the relevant national legislation that regulates PPP’s use but contains no specific evaluation of the situation or specific measures. As this problem has been addressed since 1998, and the first NAP was developed in 1986, Denmark is one of the most advanced countries in achieving success in this area.

While all the Member States’ NAPs refer to websites that contain information and raise awareness on pesticide use and their effects, Germany, UK, and Hungary do not go beyond these minimal measures. In contrast, Poland and Romania are proactive in proposing information campaigns, and Denmark, the Netherlands, and France have a more comprehensive and participatory approach. In the Netherlands and Hungary, farmers are obliged to inform their neighbors before using pesticides.

Regarding the third research question, the extent to which Directive 2009/128/EC has homogenized the approach to ameliorate the sustainability of pesticide use in particular and of agriculture in general, the answer is that in practice, the NAPs are far from homogenous. Still, no division of the old versus the new EU Member States could be identified. The UK’s NAP is the least comprehensive and detailed along the dimensions we have analyzed, a situation that has persisted from the last NAP. While it contains a complete list of indicators used to assess the evolutions in PPP usage and their impact, no clear targets and timetables are elaborated.

The NAPs of the three new EU Member States (Romania, Hungary, and Poland) treat some dimensions comprehensively and ignore others. Still, they generally do not contain indicators and quantifiable targets. Romania is one of only two Member States with planned public information measures but had no proposed concrete steps to reduce pesticides in specific areas or promote IPM and the use of low-risk products, and no mention of organic farming. By 2013, Poland had a more comprehensive approach to IPM, as it proposed measures to disseminate knowledge and proactive measures to encourage the development of an IPM production system. Meanwhile, Hungary advanced a comprehensive analysis of the sector and stressed the need for future growth.

Furthermore, Hungary’s approach to the role of low-risk products was already more advanced in 2012, as its NAP noted the importance of promoting the use of low-risk PPP, especially for minor crops. Poland went even further with a set of proposed measures to encourage the use of IPM to decrease dependency on chemical PPP. Poland had already recognized the importance of developing organic farming in 2013 and proposed measures for the sector’s encouragement.

Table 3 visually presents the differences found in the complexity with which the various NAPs treat the goals proposed in the SUD Directive. The treatments range from merely ignoring a measure to including some mentions, systematically discussing the problems, and advancing some actions to propose clearly defined steps and a timetable for their implementation. The most comprehensive approach includes a timetable for various measures and clearly defined indicators. In the last column, we have added the +s for each dimension to offer an overall evaluation. It shows considerable heterogeneity across the complexity of different EU Member States’ NAPs. While Romania and Hungary have developed the least complex NAPs, Poland has already developed a NAP more complex than that of the UK. At the opposing pole, Denmark’s NAP treats comprehensively almost all dimensions, followed by France and the Netherlands. These differences are even more relevant as the NAPs of these countries go beyond simply ticking various boxes of the SUD. The NAPs of Denmark (with its ’green growth’ policy), the Netherlands (with its ’Green Deal’), and France (with agroecology) are built around a vision of transforming agriculture as a whole and employing measurable goals. However, none of them treats all the dimensions equally comprehensively. For example, France’s agroecology vision implies a broader transformation of the approach to food production based on seven principles (a 50% reduction of PPP in two or three phases, 360-degree monitoring, developing an agroecological project, placing business at the heart of the mechanisms, working together, working at a local level, creating a positive ethos [27]. Still, it also contains some quantitative indicators and targets regarding DEPHY farms’ development [7]. It is worth stressing that after 2011, only Denmark’s pesticides sales per agricultural land have decreased, France’s have increased, while the Netherlands’ has stagnated at the highest level within the group.

## Conclusions

The current article is, to our knowledge, the first qualitative comparative analysis of EU Member State NAPs as drawn following the goals outlined in the SUD 2009/128/EC, while looking at the evolution over time of five Member State NAPs. Based on the comparative analysis of the NAPs of eight countries, we have shown that they are highly heterogeneous and vary significantly in their complexity. Although they have some common chapters, they are challenging to assess, as the depth of treatment of various dimensions differs dramatically. They do not use the same quantitative indicators or logic for proposing timetables.

Unlike other EU legislation, we reiterate that directives propose goals but leave the means to achieve them to the EU Member States. The SUD defined an overarching objective (the sustainable use of pesticides), three goals (risk reduction, promotion of IPM, low-risk alternatives to pesticides), and a set of compulsory action areas. Still, SUD proposed no quantifiable means to assess progress and no mandatory targets. Instead, each EU Member State was supposed to propose measurable objectives, targets, measures, and indicators that would allow for verifying its NAP implementation.

Regarding the first research question (RQ1), the most important conclusion from our analysis is that except for Denmark, France, and, partially, Netherlands, the analyzed NAPs do not meet the goals set in Article 4 of the SUD to "advance quantifiable objectives, targets, measures and indicators," defined as ++++ in Table 3, for most variables. Another important implication of our analysis is that while the approaches that would decrease reliance on synthetic pesticides are introduced more comprehensively in some countries’ policies to transform their agriculture, most difficulties still lie ahead.

The second research question (RQ2) investigates the incremental approach in developing NAPs from the pre-SUD to the post-SUD period. The analysis along the third research question (RQ3) brings the positive discovery that at least some of the EU’s new Member States appear to have advanced reasonably quickly in their approach to their NAP. All in all, the first-generation NAPs following the adoption of the Directive 2009/128/EC are very difficult to compare, as they lack a standard logical structure, treatment of measures, common indicators, or approach to developing timetables. Implicitly, they do not lead to a truly common approach to achieving sustainability of pesticide usage, thus improving agriculture’s sustainability.

Given that SUD contained no targets and compulsory measures, most EU MS chose the most accessible route: develop NAP’s that would discuss various issues and avoid committing to clear measures, timetables, and indicators. The second (IPM) and third (low-risk alternatives and organic agriculture) SUD goals were discussed but not operationalized. Also, most NAPs did not contain some elements deemed compulsory: timetables and targets, provisions regarding the application of IPM by all professional users by 2014, and crop and sector-specific guidelines. The conclusions of the European Court of Auditors’ and RISE Foundation reports are of no surprise, as the NAPs analysis, which represents the best-case-scenario, show that most MS did not even bother actually to plan and implement measures to achieve these goals, and the European Commission did not put any relevant pressures on them to do so.

While our analysis and EC’s 2017 reports focused on the same topic, our conclusions are sensible about the second and third SUD goals. These differences underline the added value of independently analyzing the original documents instead of using questionnaires completed by the MS. Regarding the SUD’s second goal, the European Commission’s evaluation depicted a rosy picture, indicating the presence of a mix of tools to support IPM in all MS and the availability of CAP funds to promote organic agriculture. The report was also based on a field visit in six EU MS, out of which four are also analyzed in this article (Denmark, Germany, Netherlands, Poland) [11]. The EC’s mission noted the MS’s assessment that the vast majority of growers were IPM compliant, the situation contradicted by the analysis of their NAPs and the 2020 evaluations by European Court of Auditors’ and RISE Foundation reports. Also, unexplainably, European Commission’s 2017 report contains only two mentions of low-risk alternatives (in the Netherlands case) but ignores any systematic discussion of the absence of these measures, despite that it was one of the three goals of the SUD.

Given the difficulties in comparatively assessing the harmful effects of pesticides across the EU, the 2019 calculation of the Harmonized Risk Indicators represents excellent progress. As soon as possible, detailed data for EU MS should be calculated, and clear and compulsory targets should be set. Without such comparable metrics, it would be difficult to compare different countries’ evolution or allow the potential and systematic transfer of good practices with proven positive impact.

In order to achieve a truly European approach to the sustainable use of pesticides, more uniformity in the Member States’ NAPs should be encouraged, especially in terms of proposing a set of comparable indicators to assess progress in the various areas. While our analysis does not reveal the SUD Directive’s total ineffectiveness, the main limitation is that it did not create any mechanisms to assess progress. The adoption of the 2019/782/EC Directive amending the SUD Directive (12) to speed the adoption of harmonized indicators to compare progress across the EU Member States, objectively represents the first step in this direction. While the NAPs are just plans and do not reflect all the efforts in this area, it is hardly believable that the EU Member States would go beyond the efforts they propose in these plans. To become more effective in generating a common EU approach to the sustainable use of pesticides, more standardization of NAPs should be required. This would imply asking each Member State to comprehensively treat each dimension in terms of the measures, timetable, and indicators they use and to encourage a pan-EU exchange of information, a good practices exchange mechanism, and a joint research agenda.
